# Imaging on the painful and compressed nerve: lower extremity

**DOI:** 10.1007/s00264-025-06419-1

**Published:** 2025-01-28

**Authors:** Marcelo Bordalo

**Affiliations:** Aspetar Orthopedic and Sports Medicine Hospital, Doha, Qatar

**Keywords:** MR-neurography, Peripheral nerves, Neuropathy, MRI, Ultrasound

## Abstract

Entrapment neuropathies of the lower extremity are often underdiagnosed due to limitations in clinical examination and electrophysiological testing. Advanced imaging techniques, particularly MR neurography and high-resolution ultrasonography (US), have significantly improved the evaluation and diagnosis of these conditions by enabling precise visualization of nerves and their surrounding anatomical structures. This review focuses on the imaging features of compressive neuropathies affecting the lumbosacral plexus and its branches, including the femoral, obturator, sciatic, common peroneal, and tibial nerves. Key conditions such as meralgia paraesthetica, piriformis syndrome, and tarsal tunnel syndrome are discussed, highlighting findings such as nerve thickening, T2 hypersignal, fascicular changes, and associated muscle denervation patterns. The ability to detect structural causes, including anatomical variations, fibrous bands, and space-occupying lesions, underscores the value of these imaging modalities in facilitating early diagnosis, guiding therapeutic interventions, and improving patient outcomes.

## Introduction

Entrapment neuropathies of the lower extremities are frequently underdiagnosed, as clinical examination and electrophysiological testing may lack sensitivity and reliability.

The evaluation of painful and compressed nerves in the lower extremity has been greatly enhanced by advanced imaging techniques such as MR neurography and high-resolution US. These modalities allow precise visualization of neural structures and surrounding anatomy, improving the diagnosis and characterization of compressive neuropathies. Nerves in the lumbosacral plexus, including the femoral, obturator, sciatic, common peroneal, and tibial nerves, are particularly susceptible to entrapment or compression at specific anatomical locations. Conditions such as meralgia paraesthetica, piriformis syndrome, and tarsal tunnel syndrome are common clinical presentations. This review article highlights the role of imaging in identifying nerve abnormalities, including thickening, signal changes, and associated muscle denervation patterns, which are essential for accurate diagnosis and management.

## Overview of imaging techniques

### MR Neurography

MR neurography is a specific imaging technique optimized for the study of peripheral nerves, that enables the visualization of the peripheral nerve anatomy, contours, fascicular arrangement, signal, and caliber alterations [1].

## High-resolution Ultrasound

Advancements in ultrasound (US) technology, including improvements in hardware, software algorithms, and high-frequency linear transducers (> 18 MHz), have markedly enhanced its spatial resolution, thereby improving its utility in assessing peripheral nerves. High-resolution US serves as a non-invasive and readily accessible diagnostic modality, offering critical insights for diagnosing neuropathies and monitoring treatment outcomes [2].

## Lumbosacral plexus

**Fig. 1:**
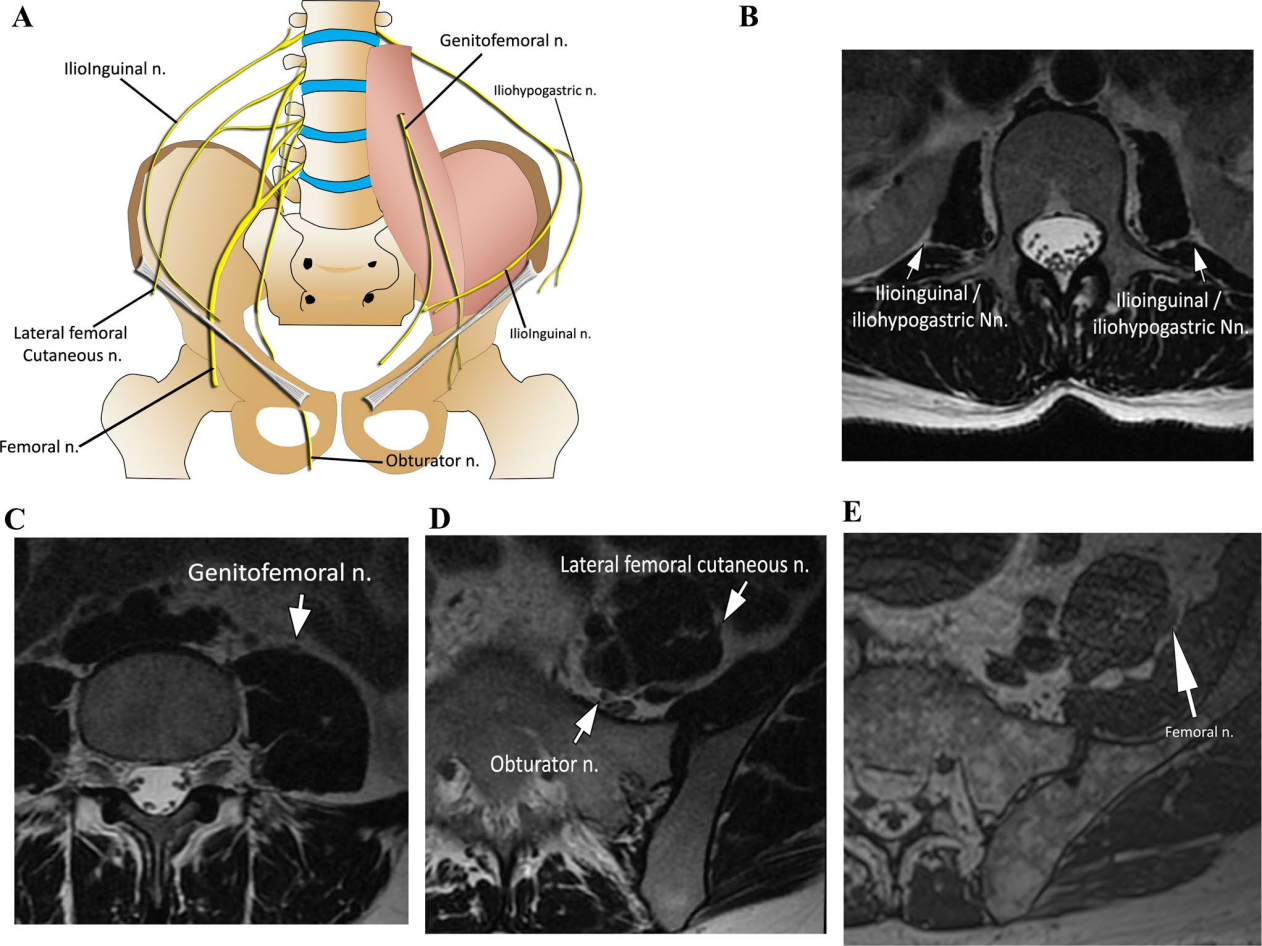
Schematic drawing of the lumbar plexus (**A**). Lumbar plexus MR anatomy. Axial T2-weighted images of the lumbar plexus at the levels of L2 (**B**), L3 (**C**), S1 (**D**) and S1/S2 (**E**) vertebral bodies. The lumbar plexus nerves are depicted in relation to the psoas muscle

The lumbar plexus nerves course around or within the psoas muscle. Axial MR images are useful in visualizing the nerves: the iliohypogastric, ilioinguinal, lateral femoral cutaneous, and femoral nerves run lateral to the psoas muscle, while the genitofemoral nerve courses anteriorly and the obturator nerve posteromedially to this muscle (Fig. 1).

Ultrasound imaging of the lumbosacral plexus has limited utility due to the deep anatomical location of the nerves, which presents challenges for effective visualization.

The sacral plexus forms the sciatic, superior gluteal, inferior gluteal, and pudendal nerves [3].

**Fig. 2:**
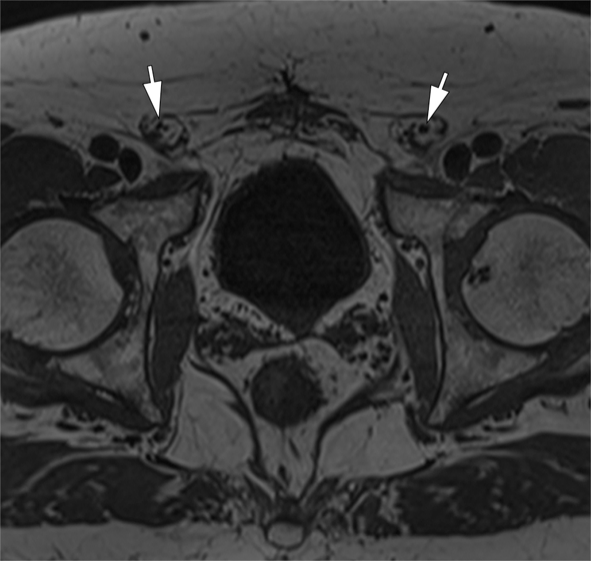
Genitofemoral nerve. Axial 3D volumetric T1-weighted image at the level of the hips demonstrates the normal genitofemoral nerves within the inguinal canals (arrows)

**The genitofemoral nerve** runs along the front edge of the psoas muscle. In males, this nerve enters the inguinal canal, and may be a cause of groin pain in athletes [4]. MRI neurography visualizes the nerve’s course from its origin at the anterior psoas to its pathway through the inguinal canal (Fig. 2).

**Fig. 3:**
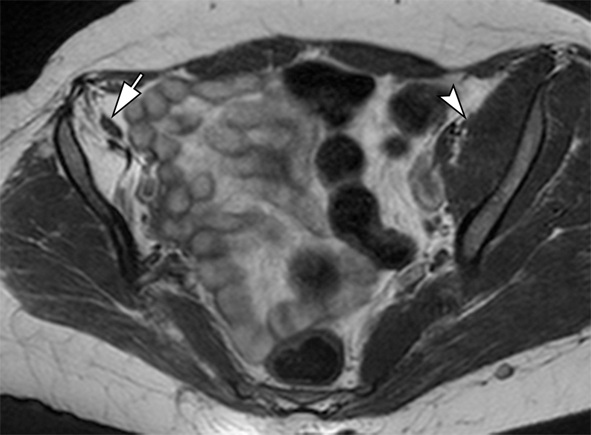
Femoral nerve lesion. A 44 year-old male with a right femoral nerve iatrogenic injury related to a hip prosthesis surgery. Axial T1-weighted image shows thickening of the right femoral nerve (arrow) and complete atrophy of the iliopsoas muscles. The left femoral nerve is normal (arrowhead)

**The femoral nerve** is located along the posterolateral border of the psoas major, extending medially toward the iliacus muscle. It exits the pelvis beneath the inguinal ligament, lateral to the femoral vessels. MRI is particularly effective in detecting abnormalities of this nerve, especially within the pelvic region (Fig. 3) [3]. 

**Fig. 4:**
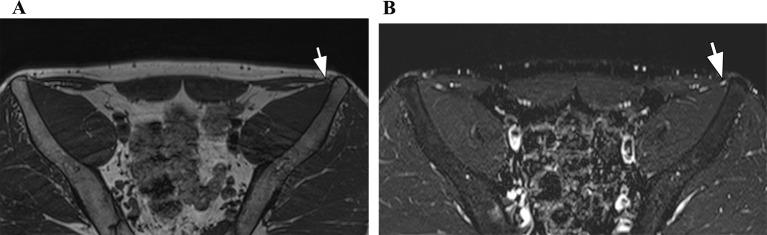
Lateral femoral cutaneous nerve injury. 49 year-old male with left meralgia paraesthetica. Axial 3D volumetric T1-weighted (**A**) and STIR (**B**) images of the pelvis show the left lateral femoral cutaneous nerve with thickening and increased signal (arrows), indicating neuropathy

**The lateral femoral cutaneous nerve** is found lateral to the psoas muscle, traveling downward along the iliacus muscle and exiting the pelvis near the anterosuperior iliac spine through or under the inguinal ligament. Compression of this nerve causes meralgia paresthetica, characterized by tingling and numbness in the upper, outer thigh. Typical causes include tight clothing, retroperitoneal masses, surgical trauma, or sartorius muscle injury near its attachment. MRI neurography and high resolution US helps identify thickening or signal changes in the nerve (Fig. 4) [3, 5, 6]. 

**Fig. 5:**
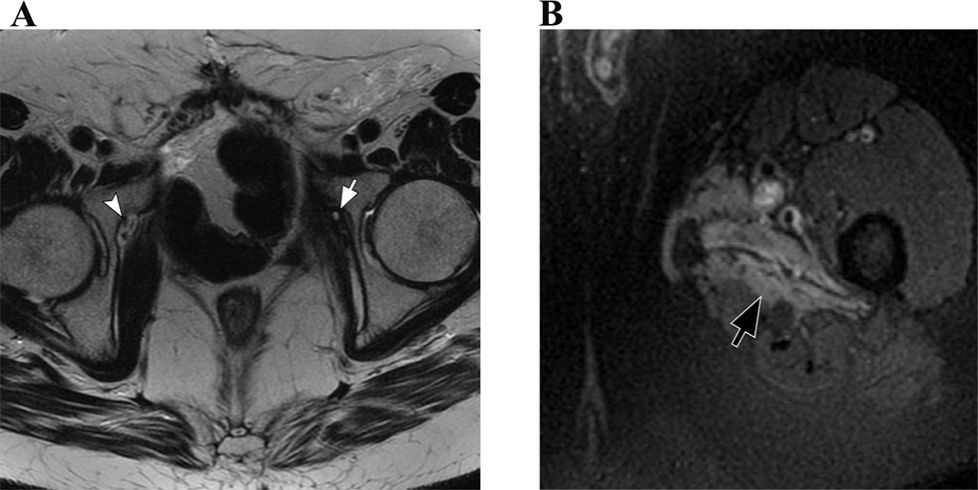
Obturator neuropathy. Patient with a previous left supraacetabular fracture 4 years ago develops left obturator neuropathy. (**A**) Axial T2-weighted image shows complete obliteration and fibrous tissue surrounding the left obturator nerve (white arrow). The right obturator nerve is normal (arrowhead). (**B**) Axial T2-weighted image with fat suppression of the proximal thigh. There is muscle edema in the left adductor muscles (black arrow), related to denervation

**The obturator nerve** runs adjacent to the posteromedial aspect of the psoas muscle and exits the pelvis through the obturator foramen. MRI findings for abnormalities in this nerve may include denervation changes in the adductor muscles and nerve thickening with increased signal intensity (Fig. 5) [7, 8]. 

**Fig. 6:**
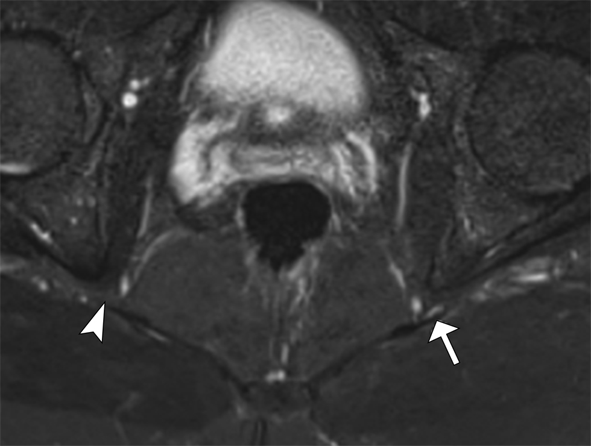
Pudendal neuropathy. 36 year-old male with left pudendal nerve symptoms. Axial 3D volumetric STIR image depicts thickening and increased signal of the left pudendal nerve in its course anterior the sacrotuberous ligament (arrow). The right pudendal nerve is normal (arrowhead)

**The pudendal nerve** is prone to compression in individuals who remain seated for extended periods or cyclists experiencing repeated saddle pressure. High-resolution MRI neurography identifies the nerve as the most posterior structure within Alcock’s canal. Advanced imaging techniques help detect abnormalities in this nerve (Fig. 6).

**Fig. 7:**
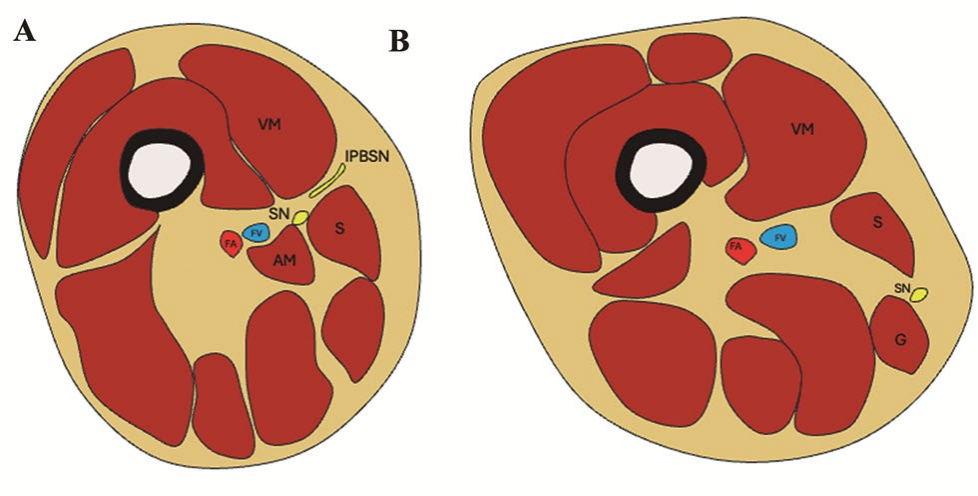
Schematic illustration of the adductor canal and saphenous nerve anatomy. (**A**) In a proximal axial section of the distal thigh, the saphenous nerve (SN) is positioned medial to the femoral artery (FA) and femoral vein (FV), located between the adductor magnus (AM) and sartorius (S) muscles. The infrapatellar branch of the saphenous nerve (IFBSN) emerges between the vastus medialis (VM) and sartorius (S) muscles. (**B**) In a more distal section, the saphenous nerve (SN) becomes more superficial as it courses between the sartorius (S) and gracilis (G) muscles

**The saphenous nerve** provides sensory innervation to the medial, anteromedial, and posteromedial regions of the lower limb, including the distal thigh, prepatellar area, medial calf, and medial aspect of the foot. It courses medially in the upper thigh, lying laterally in relation to the femoral vessels. The nerve enters the adductor canal, also known as Hunter’s canal, a musculoaponeurotic tunnel. Within the canal, it gives rise to an infrapatellar branch before continuing distally to innervate the lower leg (Fig. 7). The musculoaponeurotic structures that define the borders of the adductor canal, as well as its contents, can be readily visualized using MRI and ultrasound [9, 10].

**Fig. 8:**
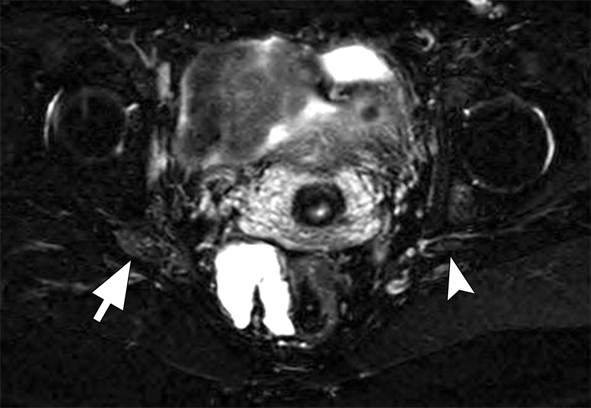
Sciatic neuropathy. Axial 3D volumetric STIR image of the pelvis. The right sciatic nerve is thickened and with increased signal and loss of the regular fascicular pattern, indicating neuropathy (arrow). Note the normal left sciatic nerve (arrowhead)

**Fig. 9:**
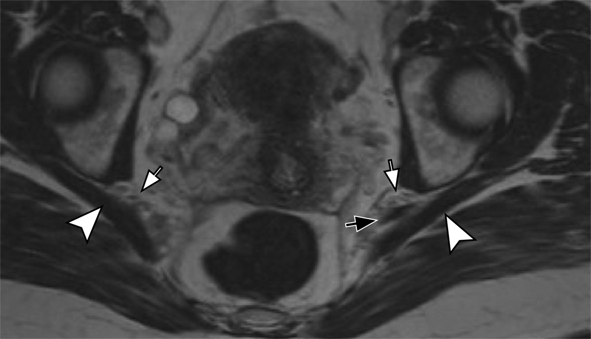
Fibrous band. A 41 year-old male with left sciatic nerve symptoms. Axial 3D volumetric T1-weighted image of the pelvis demonstrating both sciatic nerves (white arrows) and the piriformis muscles (arrowheads). There is a thick fibrous band (black arrow) posterior to the left sciatic nerve. (courtesy of Dr Denise Tokechi Amaral, São Paulo, Brazil).

**The sciatic nerve** consists of two divisions: the medial tibial and lateral common peroneal. MRI findings indicative of abnormalities include enlargement of the nerve with fluid-sensitive signal changes similar to adjacent blood vessels (Fig. 8). Mild signal alterations may occur normally, but significant abnormalities in the fascicular pattern or surrounding fat planes are diagnostic. In cases of piriformis syndrome, MRI neurography is essential for assessing the nerve’s relationship with surrounding muscles, anatomical variants, or fibrous bands (Fig. 9).

## Common peroneal nerve

**Fig. 10:**
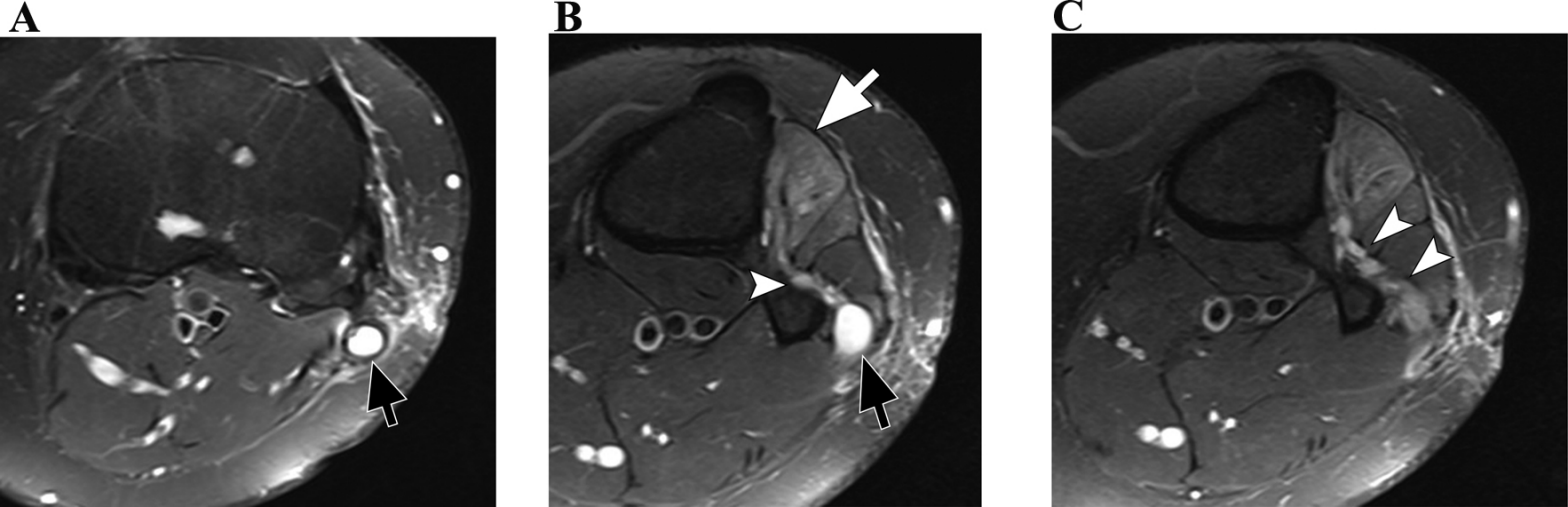
Axial T2-weighted consecutive images (**A**, **B** and **C**) of the left knee with fat suppression. There is a cyst within the common peroneal nerve (black arrows), originating from the proximal tibiofibular joint through its articular nerve branch (arrowheads). There is denervation oedema within the extensor muscles (white arrow)

The sciatic nerve splits into the tibial and common peroneal nerves, typically at the popliteal fossa. The common peroneal nerve is more vulnerable to compression near the fibular neck due to its superficial location. Intraneural ganglia originating from the tibiofibular joint may extend into the articular branch of the common peroneal nerve. MRI neurography reveals thickening or signal changes in the nerve (Fig. 10) [11, 12]. 

The superficial peroneal nerve is most commonly compressed at the lower third of the leg, where it transitions through the muscular fascia into the subcutaneous tissue. Athletes and dancers are particularly prone to this condition due to repetitive foot inversion and plantar flexion. In general, any ankle sprain resulting in loss of sensation on the dorsum of the foot should include clinical and imaging focused on the superficial peroneal nerve, as this is commonly overlooked. Ultrasound is a useful method in providing complementary diagnostic information in the common and superficial peroneal nerves compressive neuropathies [13].

## Tibial nerve

Compression of the tibial nerve in the leg or tarsal tunnel is often linked to external factors such as cysts, tumours, aneurysms, or ganglia. High-resolution MRI neurography is instrumental in identifying abnormalities and assessing the nerve’s condition in these cases [11]. 

### Collaboration between the radiologist and surgeon

Imaging of peripheral nerves requires a tailored approach by the radiologist, encompassing specific considerations for hardware, imaging protocols, acquisitions, and post-processing techniques. Collaboration with the nerve surgeon is essential for achieving an accurate diagnosis, as subtle clinical findings can guide the radiologist in identifying the underlying cause of symptoms. Furthermore, the radiologist plays a critical role in precisely localizing nerve compressions, which can greatly assist the surgeon during operative planning and procedures. Imaging also allows for the identification of anatomical variations in nerve structures, reducing the risk of intraoperative injuries and enhancing surgical outcomes.

## Summary

Advanced imaging techniques, particularly MR neurography and high-resolution MRI, are invaluable tools for evaluating nerve entrapment syndromes in the lower extremity. This chapter focuses on the imaging of key nerves within the lumbosacral plexus and their common compression sites, such as the inguinal ligament, piriformis muscle, fibular neck, and tarsal tunnel. Imaging findings include nerve thickening, T2 hypersignal, and fascicular pattern changes, along with denervation patterns in the corresponding muscles. In specific conditions such as meralgia paraesthetica, pudendal neuralgia, piriformis syndrome, and peroneal nerve entrapment imaging provides valuable information in detecting structural causes like tumors, fibrous bands, or anatomical variations. These imaging modalities enable early diagnosis and guide therapeutic interventions to improve patient outcomes.

## Data Availability

No datasets were generated or analysed during the current study.
